# A Nacre‐Like Carbon Nanotube Sheet for High Performance Li‐Polysulfide Batteries with High Sulfur Loading

**DOI:** 10.1002/advs.201800384

**Published:** 2018-04-19

**Authors:** Zheng‐Ze Pan, Wei Lv, Yan‐Bing He, Yan Zhao, Guangmin Zhou, Liubing Dong, Shuzhang Niu, Chen Zhang, Ruiyang Lyu, Cong Wang, Huifa Shi, Wenjie Zhang, Feiyu Kang, Hirotomo Nishihara, Quan‐Hong Yang

**Affiliations:** ^1^ Engineering Laboratory for Functionalized Carbon Materials Shenzhen Key Laboratory for Graphene‐based Materials Graduate School at Shenzhen Tsinghua University Shenzhen 518055 China; ^2^ School of Materials Science and Engineering Tsinghua University Beijing 100084 China; ^3^ Department of Materials Science and Engineering Stanford University Stanford CA 94305 USA; ^4^ Tsinghua‐Berkeley Shenzhen Institute (TBSI) Tsinghua University Shenzhen 518055 China; ^5^ Institute of Multidisciplinary Research for Advanced Materials Tohoku University Sendai 980−8577 Japan; ^6^ School of Chemical Engineering and Technology Tianjin University Tianjin 300072 China; ^7^ School of Marine Science and Technology Tianjin University Tianjin 300072 China

**Keywords:** carbon nanotube sheets, high sulfur loading, Li‐polysulfide batteries, Li‐sulfur batteries, nacre‐like materials

## Abstract

Lithium‐sulfur (Li‐S) batteries are considered as one of the most promising energy storage systems for next‐generation electric vehicles because of their high‐energy density. However, the poor cyclic stability, especially at a high sulfur loading, is the major obstacles retarding their practical use. Inspired by the nacre structure of an abalone, a similar configuration consisting of layered carbon nanotube (CNT) matrix and compactly embedded sulfur is designed as the cathode for Li‐S batteries, which are realized by a well‐designed unidirectional freeze‐drying approach. The compact and lamellar configuration with closely contacted neighboring CNT layers and the strong interaction between the highly conductive network and polysulfides have realized a high sulfur loading with significantly restrained polysulfide shuttling, resulting in a superior cyclic stability and an excellent rate performance for the produced Li‐S batteries. Typically, with a sulfur loading of 5 mg cm^−2^, the assembled batteries demonstrate discharge capacities of 1236 mAh g^−1^ at 0.1 C, 498 mAh g^−1^ at 2 C and moreover, when the sulfur loading is further increased to 10 mg cm^−2^ coupling with a carbon‐coated separator, a superhigh areal capacity of 11.0 mAh cm^−2^ is achieved.

In recent years, research on lithium‐sulfur (Li‐S) batteries has been prevailing because of their high theoretical energy density of 2600 Wh kg^−1^ (material based) or over 600 Wh kg^−1^ (package based), which is by far superior to that of conventional lithium‐ion batteries (LIBs).^1^, ^2^, ^3^, ^4^, ^5^, ^6^, ^7^, ^8^, ^9^, ^10^, ^11^, ^12^, ^13^ However, the intractable issues that are faced in this field have retarded the practical use of this advanced energy storage system. These issues have been comprehended as the poor electrical conductivities of sulfur and discharge products (Li_2_S_2_/Li_2_S), large volume change of sulfur (80%) upon lithiation and delithiation, and the “shuttle effect” of the intermediate lithium polysulfides (LiPSs) that leads to the quick capacity decay and lithium anode erosion.^1^, ^7^ In general, utilization of electrically conductive and porous hosts,^14^, ^15^, ^16^, ^17^, ^18^, ^19^, ^20^ LiPS blocking interlayers^21^, ^22^, ^23^, ^24^ or modified separators^25^, ^26^, ^27^, ^28^ have been the main strategies to address the above issues, especially for restraining the “shuttle effect”. Another issue is the limited sulfur loading (mostly ≤3 mg cm^−2^) that could not meet the requirements for commercial use. In this regard, utilization of 3D sulfur hosts has been reported to be appealing.^29^, ^30^, ^31^, ^32^, ^33^ However, sulfur loading and “shuttle effect” is a trade‐off. Also, the close contact between the excessive active materials and the electrically conductive matrix is highly required to realize a good electrochemical performance. Besides, it is necessary to guarantee a uniform distribution of the active material to ensure the reaction kinetics and alleviate variation of the electrode structure.^33^

Herein, we report a nacre‐like carbon nanotube sheet for high performance Li‐S batteries with a high areal sulfur loading. Specifically, conductive carbon nanotube (CNT, dispersed by polyvinylpyrrolidone, PVP) was used as the building blocks. By a unidirectional freeze‐drying (UDF) approach and the subsequent mechanical compression, we have realized the construction of the nacre‐like counterpart (denoted NS, short for the nacre‐like sheet). The in‐layer and interlayer space of the conductive CNT layers enable the accommodation of a large amount of sulfur (in the form of LiPSs, as will be described later) while ensuring a highly conductive network from the compact configuration. Besides, the amphiphilic PVP, which is originally used as the dispersing agent for the preparation of CNT‐based NS, strongly interacts with LiPSs to help suppress the unfavorable migration of LiPSs during charge/discharge processes. The NS has enabled an equivalent areal sulfur loading of 5 mg cm^−2^, a discharge capacity of 1236 mAh g^−1^ at 0.1 C, a high rate discharge capacity of 498 mAh g^−1^ at 2.0 C, and outstanding cycling stability with a Coulombic efficiency of over 99.9% on average. Moreover, when the equivalent areal sulfur loading was further increased to 10 mg cm^−2^ with the combination of a carbon‐coated separator, a high areal capacity of up to 11.0 mAh cm^‐2^ was achieved.

The nacre of abalones is well known to have a lamellar structure consisting of layers of aragonite and protein (**Figure** **1**a). The ordered brick‐and‐mortar arrangement of inorganic and organic layers renders a high mechanical strength and has triggered the explosion on artificially synthesizing similar counterparts.^34^ While previous works have focused mainly on the mechanical properties of those counterparts, the compact configuration of the structure has inspired us to develop a cathode with a similar structure for Li‐S batteries with a high sulfur loading. The concept here is to design a nacre‐like construction in which a large amount of sulfur is compactly embedded (Figure 1b).

**Figure 1:**
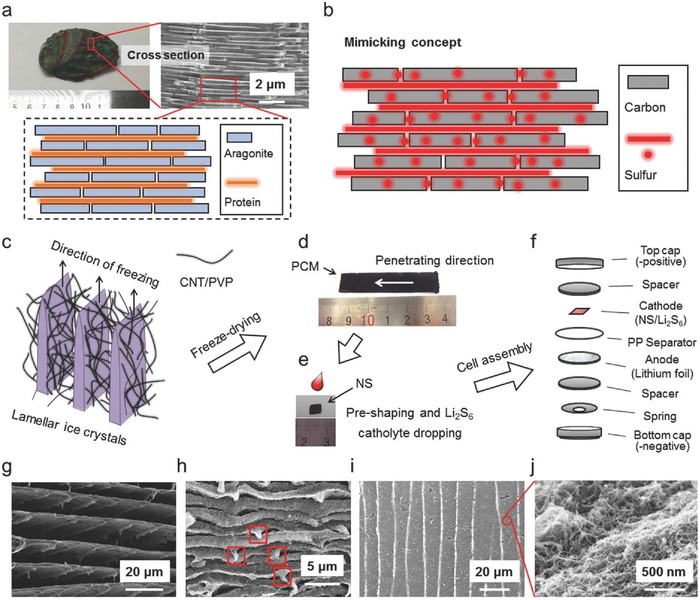
Nacre structure of an abalone and the concept of mimicking, together with schematic representation of the preparation of PMC, NS, electrochemical cells of Li‐sulfur batteries, and structural characterizations of PMC and NS. a) Digital image of the abalone shell (*Haliotis* diversicolor supertexta) from Wenzhou, a southern city of China (left), SEM image of the nacre of the shell (middle), and structural model for a typical nacre. b) The concept of mimicking the nacre structure with carbon and sulfur. c) Illustration of the ice‐crystal growth during the unidirectional freezing of the CNT aqueous dispersion that was used to prepare the PCM. d) An optical image of the PCM. A white arrow shows the direction of its penetrating channels. e) An optical image of the NS. As shown here, Li_2_S_6_ catholyte is dropped on the NS to form the cathode. f) Schematic representation for assembling the corresponding Li‐polysulfide battery using the NS as the cathode. g) SEM image of the PCM for its cross section along the thickness direction. h,i) SEM images of the NS for its cross sections along h) the thickness direction and i) the length direction. The marked areas indicate the maintained rib‐like protrusions after compression. j) High‐magnification image of panel (i).

In order to achieve such a structure, a porous CNT monolith (PCM) with interval interspace was first prepared through the UDF approach.^35^, ^36^ The UDF is a flexible technique through which a series of ordered structures can be prepared. In order to achieve the nacre‐like counterpart for Li‐S batteries, we paid efforts on determining the precursor dispersion and finalized it as a CNT system.^37^ Typically, an aqueous dispersion of CNT (≈5 wt%) with PVP (≈1 wt%) as a dispersing agent was loaded into a one‐end‐closed cuboid polytetrafluoroethylene mold (the inner space is 3 × 12 × 70 mm), and the mold was unidirectionally immersed into liquid nitrogen at a constant velocity of 50 cm h^−1^. During the unidirectional freezing process, phase separation took place, where water solidified to form lamellar ice crystals and CNT/PVP was expelled to their interspace (Figure 1c).^38^ PVP remained on the CNT surface (Figure S1, Supporting Information) and functioned as an anchor of LiPSs, as shown later. By freeze drying, the ice crystals were removed, yielding the PCM molded into a cuboid (Figure 1d). The PCM thus obtained was cut and compressed to be NS that accommodates Li_2_S_6_ catholyte (Figure 1e) and further assembled into coin cells (Figure 1f). Scanning electron microscopy (SEM) of the cross section of the PCM along the thickness direction reveals a lamella‐like micromorphology with an average interspace of ≈10.7 µm (Figure 1g). It should be noted that there are rib‐like protrusions on each of the lamellar layers, and some of the protrusions interconnect the layers, ensuring the structural integrity of the monolith (Figure S2, Supporting Information). After compression, the PCM was turned into a much more compact structure that resembles the nacre structure and these protrusions can be maintained and only have a little deformation as shown in Figure 1h. The average interspace in the NS was decreased to ≈320 nm. Figure 1i shows a wide‐range image of a lamella layer, showing its long‐range continuity. The long‐range continuity is ascribed to the large plate‐like ice crystals that were formed during the unidirectional freezing process. This differs from the freezing that takes place in a refrigerator, where ice crystals with random shapes are formed and a slack structure with much worse structural integrity was obtained after freeze drying (Figure S3, Supporting Information). By compression, a counterpart sample, randomly porous CNT sheet (RS), was prepared (Figure S4, Supporting Information). The individual layer in the NS is composed of highly twisted and interlaced CNT/PVP (Figure 1j), yielding a specific surface area of 132 m^2^ g^−1^ and a large pore volume of 1.3 cm^3^ g^−1^ (Figure S5, Supporting Information). Such a pore volume is theoretically large enough to accommodate a substantial amount of sulfur within (≈3.1 mg sulfur per mg NS), which fundamentally guarantees a high sulfur loading for the NS electrode.

As shown in Figure 1e, sulfur was introduced into the NS in the form of Li_2_S_6_, which was reported to be superior in the Li‐S redox reaction kinetics.^33^ The preliminary tests for Li_2_S_6_ catholyte penetrating demonstrated that the NS is more capable of accommodating Li_2_S_6_ catholyte than the RS (Figure S6, Supporting Information). This is ascribed to the continuous lamella microgaps that were generated from the ice templates, which functions better than the random RS structure and is more capable of accommodating catholyte. What is worth noting is that a pure CNT membrane that does not contain PVP was hardly wetted by the Li_2_S_6_ catholyte (Figures S6 and S7, Supporting Information).

An equivalent areal sulfur loading of 5 mg cm^−2^ (calculated from the amount of Li_2_S_6_ added) was adopted to assemble the coin cells. At the beginning, the cells were subjected to precharging (delithiation at 0.1 C up to 3.0 V (Figure S8, Supporting Information), during which the Li_2_S_6_ in the cathode was oxidized to S/Li_2_S_8_.^29^ The higher charge capacity for the NS included cell indicates that the NS has accommodated a larger amount of catholyte than the RS and the CNT membrane. **Figure** **2**a gives the subsequent galvanostatic charge/discharge profiles of the NS electrode at the rates from 0.1 to 2.0 C. Two clear plateaus are identified in the discharge (lithiation) curves, in which the upper plateau corresponds to the reduction of S/Li_2_S_8_ into LiPSs, while the lower plateau is ascribed to the reduction of LiPSs into Li_2_S_2_/Li_2_S.^29^ Even at a high rate of 2.0 C, the two plateaus are still remained, showing good kinetics of the NS electrode. Note that when we tried to introduce elemental sulfur into the NS, the systems showed lower capacity and reaction kinetics than that using catholyte system (Figure S9, Supporting Information). This mainly comes from the aggregation of elemental sulfur in the macropores of NS, leading to weak contact between sulfur and the carbon substrate and low reaction activity.^1^ The behavior of the RS electrode is basically similar (Figure 2b), whereas its capacity is not as high as that of the NS electrode. This proves the inferior capability of the RS electrode in accommodating catholyte, showing low capacities at even lower rates (Figure S10, Supporting Information). Figure 2c shows the rate performances of the NS and RS electrodes. The NS electrode delivers discharge capacities of 1236, 1156, 1092, 1004, 958, and 498 mAh g^−1^ at rates of 0.1, 0.2, 0.3, 0.5, 1.0, and 2.0 C, respectively, showing a good rate performance. These values are among the best of high‐sulfur loading cathodes reported (Table S1, Supporting Information). For the RS electrode, the corresponding values are 878, 775, 641, 580, 419, and 299 mAh g^−1^. The sharp drop of discharge capacity from 1.0 C to 2.0 C (≈48% that of the value at 1.0 C) for the NS electrode is possibly due to the major in‐plane ion transport path that would hinder the transport of ions at high rates, as is also mentioned later. It should be noted that at 0.1 C, the NS electrode delivers an areal discharge capacity of ≈6.2 mAh cm^−2^, which is much higher than those electrodes prepared with conventional scrape coating approach.^5^

**Figure 2:**
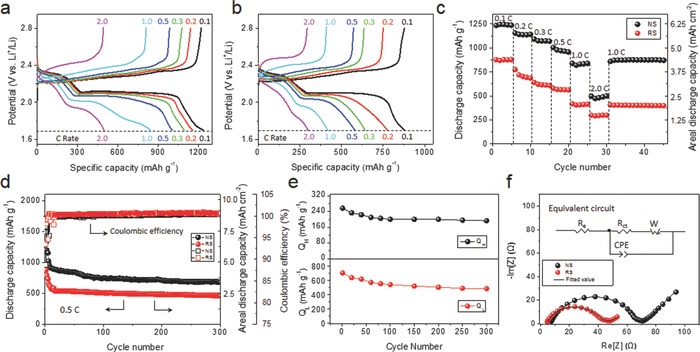
Electrochemical characterizations for the NS‐ and RS‐based cathodes. a,b) Galvanostatic charge/discharge profiles at rates from 0.1 to 2.0 C for a) the NS‐based and b) the RS‐based cathodes. c) Rate performance from 0.1 to 2.0 C for the two cathodes. Note that data for the activation cycles are not included. d) Cycling performance tested at 0.5 C for the two cathodes. Note that the initial five cycles refer to the activation cycles which include two cycles at 0.1 C and three cycles at 0.2 C. e) *Q*_H_ and *Q*_L_ values through cycling of the NS cathode. Note that data for the activation cycles are not included. f) EIS data with the fitted equivalent circuit for the two electrodes before cycling.

The cycling performance of the NS and RS electrodes was tested at 0.5 C (Figure 2d). The NS electrode delivers a discharge capacity of 1020 mAh g^−1^ (at 0.5 C, areal capacity: 5.1 mAh cm^−2^) at the initial cycle and retained 676 mAh g^−1^ (areal capacity: 3.4 mAh cm^−2^) after 300 cycles. Noteworthy is that the initial 100 cycles have involved the major capacity loss of 279 mAh g^−1^, which takes up ≈81.1% of the total capacity loss throughout the 300 cycles. The slope for the latter 200 cycles resembles that of the RS (see Figure S11 in the Supporting Information for expanded curves) indicates that the intrinsic properties of CNT/PVP enable a long‐term cyclic stability for the corresponding Li‐S batteries. Nevertheless, the RS delivers a much lower discharge capacity of only 454 mAh g^−1^ after 300 cycles, showing the advantage of the NS electrode. In a catholyte system, the excessive polysulfide that could not be constrained in the cathode started to shuttle to the anode once the cell was assembled, and a passivation layer will be formed in the initial few cycles on the lithium metal which hinders the further reduction of LiPSs onto the lithium surface. Thus, the Coulombic efficiency in the catholyte system quickly stabilizes to reach an equilibrium level, and during the long cycling, both NS and RS accordingly showed a similarly stable Coulombic efficiency. Note that after stabilization, the Coulombic efficiency for both the NS and RS electrodes is slightly higher than 100% (see Figure S12 in the Supporting Information for expanded curves). This likely comes from the reutilization of the residual LiPSs in the electrolyte during the charging process. Since the RS contains a higher amount of LiPSs in the electrolyte in the assembled cell as the RS cannot hold the high sulfur loading, it shows a slightly higher Coulombic efficiency than that of the NS. It should be noted that PVP also played an important role in the long‐term cyclic stability of the NS electrode. The CNT membrane, which is free of PVP, has shown a lower cyclic stability and Coulombic efficiency (≈99.2% on average) compared to the NS and the RS (Figure S13, Supporting Information). This comes from the effect of PVP, which provides anchoring sites for soluble polysulfides during the charge/discharge process.

The cyclic stability of the NS electrode was further investigated with the change of upper‐plateau discharge capacity (*Q*_H_) and lower‐plateau discharge capacity (*Q*_L_) (see Figure S14 in the Supporting Information for better understanding of *Q*_H_ and *Q*_L_). As can be found in Figure 2e and Figure S14 (Supporting Information), both *Q*_H_ and *Q*_L_ experienced a slow decrease with the cycling. Even with the high areal sulfur loading, the *Q*_H_ changes from the initial 257 to 194 mAh g^−1^ after 300 cycles, showing a high retention of 75.4%. This indicates that the LiPSs involved in the electrochemical process were efficiently confined within the NS matrix.^29^ The *Q*_L_ value is related to the capability of the transformation from LiPSs to poorly soluble sulfides.^39^ For the NS electrode, the *Q*_L_ value starts from 701 mAh g^−1^ and becomes 482 mAh g^−1^ after 300 cycles, giving a retention of 68.8%. The relatively high retention of *Q*_L_ reflects the high capability for the transformation reaction involved and good electrochemical reversibility. Figure 2f shows the Nyquist plots for the NS and RS electrodes. The plots are featured with a depressed semicircle at the high frequency region and an inclined line at the low frequency region, and they are fitted with an equivalent circuit depicted in Figure 2f, where *R*_e_, *R*_ct_, and *W*_o_ represent the internal resistance of the cell, the charge‐transfer resistance and Warburg impedance, respectively.^40^ The values of *R*_ct_ for the NS and RS electrodes were fitted to be 62.5 and 36.1 Ω, respectively. This is likely to be due to the more interconnected porous structure of the RS, which facilitates both electron and ion transport, thus rendering a smaller *R*_ct_ for the RS electrode. However, due to the inferior capability of accommodating Li_2_S_6_ catholyte and restraining LiPS in the RS electrode, the actual sulfur amount in the RS electrode is much smaller than in the NS electrode. As a result, the true current densities of the RS included cells become obviously higher than the set values, which results in the larger overpotentials than that of the NS included cells (Figure 2a,b), although the former has a smaller *R*_ct_ (Figure 2f). The CNT membrane turned out to have an even larger *R*_ct_ of 91.7 Ω (Figure S15, Supporting Information) and is possibly caused by its much more compact structure. The Warburg impedance *W*_o_ is caused by the diffusion of the LiPSs within the cathode.^41^ The smallest *W*_o_ of the NS (Table S2, Supporting Information) indicates the strongest ability in restricting LiPSs within its matrix.

In order to further increase the energy density of the whole system, the areal sulfur loading was increased from 5.0 to 7.5 mg cm^−2^ and 10 mg cm^−2^ by adding more Li_2_S_6_ catholyte in the cell assembling. The separator was modified with a thin layer of carbon coating^42^ for better confining the LiPSs within the cathode. **Figure** **3**a gives the rate performance of the NS electrodes with an areal sulfur loading of 7.5 mg cm^−2^ (denoted NS‐7.5) and 10 mg cm^−2^ (denoted NS‐10). At rates from 0.1 to 2.0 C, both NS‐7.5 and NS‐10 electrodes show considerably high discharge capacities. Especially, NS‐10 delivers an areal discharge capacity of ≈11.0 mAh cm^−2^, which is among the highest values that have been reported so far.^5^ The galvanostatic charge/discharge profiles show that both the NS‐7.5 and NS‐10 electrodes retain two plateaus even at 2.0 C, showing their good kinetics despite the high sulfur loading (Figure S16, Supporting Information). Additionally, the voltage hysteresis (denoted Δ*E*) for the NS‐10 electrode is slightly higher than that of the NS‐7.5 electrode because of the higher sulfur loading that has led to a higher degree of polarization (Figure S17, Supporting Information). In a long‐term cyclic test at 0.5 C, the NS‐10 electrode delivers an initial discharge capacity of 789 mAh g^−1^ (areal discharge capacity of ≈7.8 mAh cm^−2^) and stabilized at ≈610 mAh g^−1^ (areal discharge capacity of ≈6.1 mAh cm^−2^) after 500 cycles, showing a considerably high retention of 77.8% (Figure 3b). The Coulombic efficiency is slightly higher than the cell with 5 mg cm^−2^ sulfur loading and this might be due to the higher amount of LiPSs in the system (Figure S18, Supporting Information).

**Figure 3:**
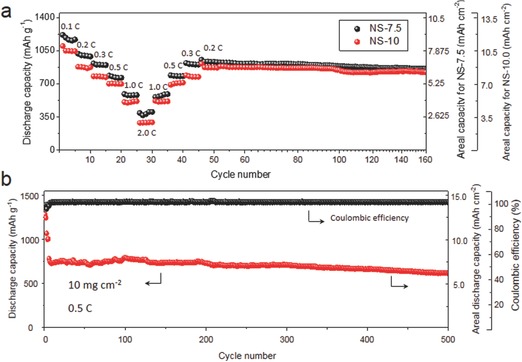
Electrochemical characterizations for the NS‐based cathodes with high areal sulfur loadings. a) Rate performance from 0.1 to 2.0 C for the NS‐7.5 and NS‐10 electrodes. Note that data for the activation cycles are not included. b) Long‐term cycling test for the NS‐10 electrode. Note that the initial five cycles refer to the activation cycles which include two cycles at 0.1 C and three cycles at 0.2 C.

The advantages for the NS cathode‐based lithium polysulfide batteries are assumed as follows. For the first and for the most, a high areal loading of sulfur can be realized by the macropores in the NS and the layer‐by‐layer nacre‐like structure that has a widespread domain size. The consistent individual layer in the NS allows a more intimate contact between sulfur and the NS substrate.^43^ Second, the integral nacre‐like CNT structure functions as a highly conductive network and also affords a mechanical stability to buffer the volume change of sulfur during cycling. Third, the amphiphilic PVP naturally remained from the preparation process of the NS improves the wetting ability of the electrode and increases the affinity of carbon surface with the polar LiPSs, thus rendering improved reaction kinetics and cyclic stability. Finally, the lamella structure provides large space for volume changes of active materials during the electrochemical processes.

To further understand the mechanism of the NS in achieving the superior electrochemical performance, a coin cell was disassembled before cycling for the energy‐dispersive X‐ray spectrometer (EDX) mapping analysis, and the result is shown in **Figure** **4**a–e. Li_2_S_6_ is confirmed to be homogeneously distributed within the whole NS matrix at the initial stage (Figure 4e). After 300 charge–discharge cycling cycles, the lamella‐like morphology of the NS was well retained. This can be ascribed to the morphological merit of the NS electrode in which mechanically tough lamella sheets consisting of the CNT network are periodically integrated through microgaps. Moreover, the EDS mappings show a homogenous distribution of sulfur (Figure S19, Supporting Information), which well verifies that PVP can anchor LiPSs and/or Li_2_S/Li_2_S_2_.^44^ When tested in a LiNO_3_‐free system, the Coulombic efficiency is over 100% because of the reaction of electrolyte species and Li foil. Nevertheless, cyclic stability is good, further supporting the positive role of PVP (Figure S20, Supporting Information). The binding energy between the carbonyl groups on PVP and the Li*_x_*S compounds is much higher than that between the bare carbon surface and the Li*_x_*S compounds. As is depicted in Figure 4g, LiPSs are attracted to the carbon surface through the polar interaction with oxygen atoms in PVP molecules. The interaction is strong and LiPSs can transform into other species without easily detaching off. This is further confirmed by the slighter morphology change and the less S content of the lithium metal surface corresponding to the NS electrode after cycling (Figure S21, Supporting Information). Also worth mentioning is that PVP has been reported to be partially dissolved in the electrolyte and aid the formation of a more efficient solid electrolyte interface layer, which helps restrain the LiPS shuttling and improve the cyclability.^45^

**Figure 4:**
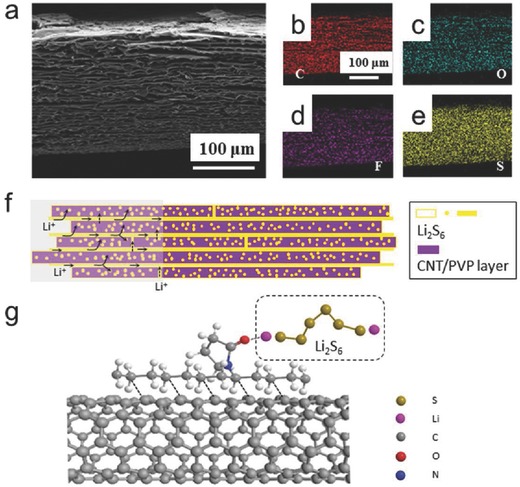
Investigations of the mechanism of the superior electrochemical performance of the NS electrode. a) SEM image of the NS electrode after catholyte introduction. b–e) Elemental distributions of b) C, c) O, d) F, and e) S by EDX. f) Schematic diagram of the NS electrode and the catholyte within for the understanding of the mechanism of the electrochemical processes. The electrolyte is not shown in the schematic representation for better understanding. g) Schematic representation showing the interactions between CNT and PVP, and between PVP and LiPSs (Li_2_S_6_ is used as an example here).

From a practical point of view, we have envisaged the potential of further increasing the areal loading of sulfur for higher energy density on a device level. One possible way is to increase the thickness of the NS electrode. However, the extended path for ion diffusion has caused conspicuous capacity loss (Figure S22, Supporting Information). Another way is to increase the number of interspace by decreasing the thickness of the individual layers of the NS. This can be achieved by adjusting the parameters that are involved in the UDF process. For example, by decreasing the concentration of the aqueous CNT dispersion, a much thinner layer could be obtained (Figure S23, Supporting Information). Another important issue for the practical application is the electrolyte/sulfur (E/S) ratio. The high E/S ratio inevitably offsets the advantage of the high theoretical capacity of sulfur and leads to the decrease of the energy density of a Li‐S battery. In this work, the corresponding E/S ratios at the sulfur loadings of 5, 7.5, and 10 mg cm^−2^ are ≈17, 16, and 11 µL mg^−1^, respectively. The E/S ratio is basically determined by the concentration of the catholyte, that is, the solubility of the polysulfide. It is required to decrease this value to a great deal as in a catholyte system in the future work.^46^

In summary, we have designed a nacre‐like sheet (NS) for Li‐S batteries with high sulfur loading by the UDF approach. The compact‐layered configuration of the NS has realized a highly conductive network even with a high sulfur loading. With an areal sulfur loading of 5.0 mg cm^−2^, the NS electrode has realized a high discharge capacity of 1236 mAh g^−1^ at 0.1 C, and a long‐term electrochemical stability at 0.5 C (initial discharge capacity of 1020.2 and 675.9 mAh g^−1^ retained after 300 cycles). When the areal sulfur loading was increased to 10 mg cm^−2^, an initial discharge capacity of 783.8 mAh g^−1^, and a discharge capacity of ≈610 mAh g^−1^ after 500 cycles was achieved, showing a high retention of 77.8% with the help of a carbon‐coated separator. Our work demonstrates a rational design of a nacre‐inspired configuration for high‐performance Li‐polysulfide batteries with a high areal sulfur loading and might also be of interest to other energy storage systems.

## Conflict of Interest

The authors declare no conflict of interest.

## Supporting information

Supplementary
