# Supplementary material for: A Nacre‐Like Carbon Nanotube Sheet for High Performance Li‐Polysulfide Batteries with High Sulfur Loading
Source: Adv Sci (Weinh). 2018 Apr 19;5(6):1800384. doi: 10.1002/advs.201800384 (PMC6010878; doi:10.1002/advs.201800384)
Supplement: Supplementary file 1 — Supplementary [file ADVS-5-1800384-s001.pdf]

Copyright WILEY-VCH Verlag GmbH & Co. KGaA, 69469 Weinheim, Germany, 2018.

ADVANCED SCIENCE
Open Access

Supporting Information

for *Adv. Sci.,* DOI: 10.1002/advs.201800384

A Nacre-Like Carbon Nanotube Sheet for High Performance Li-Polysulfide Batteries with High Sulfur Loading

Zheng-Ze Pan, Wei Lv, Yan-Bing He, Yan Zhao, Guangmin Zhou, Liubing Dong, Shuzhang Niu, Chen Zhang, Ruiyang Lyu, Cong Wang, Huifa Shi, Wenjie Zhang, Feiyu Kang, Hirotomo Nishihara,* and Quan-Hong Yang**

Copyright WILEY-VCH Verlag GmbH & Co. KGaA, 69469 Weinheim, Germany, 2013.

Supporting Information

A nacre-like carbon nanotube sheet for high performance Li-polysulfide batteries with high sulfur loading

Zheng-Ze Pan, Wei Lv, Yan-Bing He, Yan Zhao, Guangmin Zhou, Liubing Dong, Shuzhang Niu, Chen Zhang, Ruiyang Lyu, Cong Wang, Huifa Shi, Wenjie Zhang, Feiyu Kang, Hirotomo Nishihara*, Quan-Hong Yang**

Methods:

Preparation of nacre-like carbon nanotube sheet (NS). First, a porous carbon nanotube (CNT, multi-walled) monolith with unidirectionally penetrating microchannels (PCM) was prepared using a unidirectional freeze-drying (UDF) method that we have reported before[S1,2]. An aqueous CNT dispersion (Model number: NTP2021, CNT content: ca. 5.0 wt%, polyvinyl pyrrolidone (dispersant) content: ca. 1.0 wt%, Shenzhen Nanotech Port Co., Ltd.) was loaded into a self-designed cuboid container made of polytetrafluoroethylene. The container has a hollow shape, and one end is closed. The external and internal dimensions are 10x16x120 mm and 3x12x70 mm, respectively. The CNT dispersion inside the PTFE container was then frozen unidirectionally at a constant immersion speed of typically 50 cm h^{-1} into liquid nitrogen (-196 °C), lengthwise. Note that all the UDF-derived samples in this work were prepared under the above conditions unless otherwise noted. After the unidirectional freezing finished, the frozen sample was subjected to the freeze-drying process at -10 °C for 1 day, then at -5 °C for 1 day, and finally at 0 °C for 1day. Such a gradient temperature setting is used to ensure the freezing state of ice crystals and avoid the possible structure change of the ice template as well, thus guaranteeing the structural stability of the resulting monolith. The PCM thus obtained was a plate-like black monolith. Note that the temperature gradient along the immersing direction is inevitably perturbed from the vaporization of liquid nitrogen, and the pseudosteady state growth of ice crystals takes place from about 50 mm above from the bottom of the PTFE container[S3]. Accordingly, only the above part was used for structural continuity. The NS was achieved by shaping and compressing the as-obtained PCM with two stainless steel plates.

Preparation of CNT membrane. A certain amount of CNT powder (Model number: MWNT-10, >97%, Shenzhen Nanotech Port Co., Ltd.) was dispersed in ethanol with a powerful ultrasonic homogenizer (Sonics & Materials Inc., VCX-500). The dispersion was

then diluted with further adding deionized water and ultrasonic processing. Afterward, the CNT dispersion was filtered with a hydrophilic filter membrane. After drying up under ambient condition, the CNT membrane readily peeled off from the filter membrane as a self-standing sheet. The areal mass of the CNT membrane was about 4 mg cm^{-2}.

Preparation of randomly porous carbon nanotube sheet (RS). First, the above mentioned CNT dispersion (Model number: NTP2021, CNT content: ca. 5.0 wt%, polyvinyl pyrrolidone (dispersant) content: ca. 1.0 wt%, Shenzhen Nanotech Port Co., Ltd.) was loaded into a self-designed cuboid container made of polytetrafluoroethylene (PTFE). Then the container containing CNT dispersion was put inside a refrigerator (-24 °C) over night to solidify the dispersion. Afterward, the frozen sample was subjected to the freeze-drying process at -10 °C for 1 day, then at -5 °C for 1 day, and finally at 0 °C for 1day, yielding a black bulk. The bulk was then shaped and compressed to give the RS.

Preparation of 1.0 M polysulfide (Li_2S_6) catholyte. In order to prepared the 1.0 M polysulfide (Li_2S_6) catholyte, an appropriate amount of sulfur (nano particles, DeKeDaoJin Co., Ltd.) and Li_2S (99.9%, Alfa Aesar Co. Ltd.) was added into the blank electrolyte for lithium-sulfur batteries (1.0 M bistrifluoromethanesulfonimide lithium salt (LiTFSI) in 1,3 – dioxolane (DOL) : dimethoxyethane (DME) =1:1 Vol% with 2.0% $LiNO_3$, LS-009, DoDoChem Co., Ltd.). After extensive stirring the above system at 70 °C for 12 hour, a 1.0 M Li_2S_6 solution with brownish-red color was obtained.

Li_2S_6 catholyte permeating tests. NS, RS, and CNT membrane were cut and shaped into rectangular sheets with an area of 0.25 cm^2. For NS and RS, the areal mass used was about 6.0 mg cm^{-2} (with a thickness of ca. 130 μm). These samples were kept overnight in a vacuum oven at 60 °C before they were transferred into an Ar-filled glove box. NS, RS and CNT membrane were then placed on a filter paper, after which about 13 uL Li_2S_6 catholyte (1.0 M) was dropped into each sheet. Shortly after, these sheets were moved away from the original positions to check the condition of the filter paper underneath.

Materials characterizations. SEM and TEM observations were performed on a SUPRA®55 (ZEISS, Germany) and a Tecnai G2 F30 (FEI, USA), respectively. As for the sample preparation for cycled electrodes, we first rinsed the electrodes with DME to remove the residual polysulfides. We then performed the observation after drying. Nitrogen adsorption-desorption tests were conducted using a Belsorp-mini II (MicrotracBEL Corp., NIKKISO Group, Japan) at 77 K.

Electrochemical cell assembly. The NS, RS and CNT membrane were cut and shaped into rectangular sheets with an area of 0.25 cm^2. They were then assembled into Li/dissolved polysulfide batteries in an Ar-filled glove box with a fashion of coin cell (CR2032 type). The final sealing pressure was determined as ca. 50 Mpa, which accordingly is the pressure under which all the electrodes were pressed. The mass of each sheet used was 1.0-1.5 mg for the NS and RS. For the assembly of cells with a sulfur loading of 5.0 mg cm^{-2}, the commercially available PP separator Celgard 2500 was placed on the lithium-metal foil anode, followed by adding 15 uL blank electrolyte. After then, the NS, RS or CNT membrane was placed on the top of the separator, with adding 6.5 uL of the 1.0M Li_2S_6 catholyte into each sheet. For the assembly of cells with a sulfur loading of 7.5 and 10.0 mg cm^{-2}, a carbon-coated separator[S4] (the weight of the carbon layer was about 0.5 mg) was placed onto the lithium-metal foil anode, after which 20 and 25 uL blank electrolyte was added. After then, the NS was placed on the top of the separator, with adding 9.8 and 13 uL of the 1.0 M Li_2S_6 catholyte into each sheet. The role of the carbon coating on the separator is to help further suppress the LiPS shuttling to achieve a high stability, thus helping achieve a high sulfur utilization and cyclic stability under the much higher sulfur loading. It should be noted that the active material was converted to an equivalent amount of sulfur instead of Li_2S_6 for better comprehending the performance. The NS sheets used for loading 5.0 mg cm^{-2} sulfur were 1.0-1.1 mg in weight (with a thickness of ca. 130 μm), giving an equivalent sulfur content of ca. 54.0 wt% in the cathode. In the case of 7.5 mg cm^{-2} and 10.0 mg cm^{-2} sulfur loading, an equivalent sulfur

content of about 60.0 wt% was achieved (the corresponding NS electrode used has a thickness of ca. 200 μm).

Electrochemical measurements. All the cells related were confirmed to have an initial open-circuit voltage of ca. 2.3 V. Galvanostatic cycling and rate tests were conducted on a LAND CT2001 battery program controlling system at around 25 °C. The corresponding programs were set based on the theoretical capacity of sulfur (1675 mAh g^{-1}) and the corresponding areal loading of sulfur. As an example, for the NS with an areal sulfur loading of 5 mg cm^{-2}, 0.1 C and 2 C correspond to currents of ca. 0.21 mA and 4.19 mA, respectively. It is worth noting that the cathodes were first pre-charged up to 3.0 V (vercius Li/Li^+), and then charge/discharge measurements were performed in a potential window of 1.7–2.8 V. Electrochemical impedance spectroscopy measurements were performed on the cathodes with the same VMP3 electrochemical workstation in the frequency range of 1 MHz to 0.1 Hz with a 5 mV AC oscillation at the open-circuit potential.

Supplementary Figures

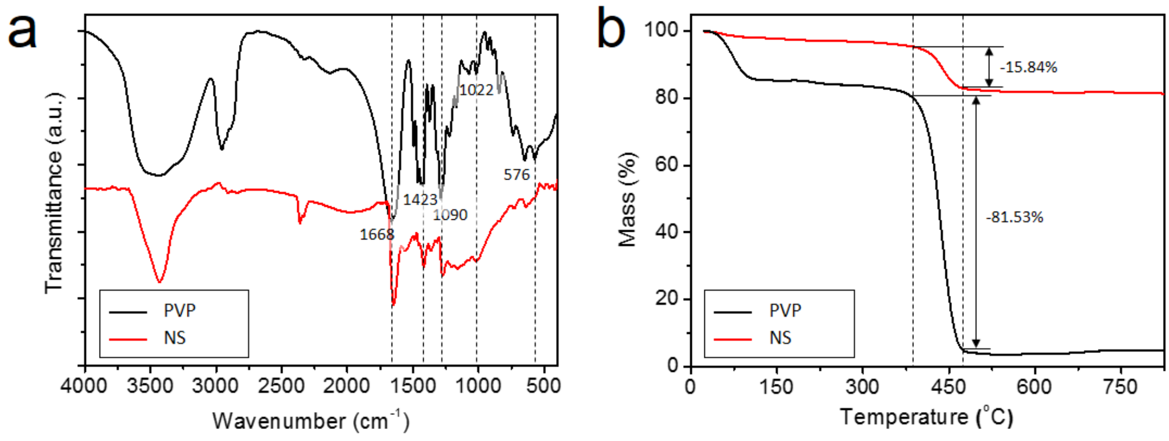

Figure S1. (a) FTIR and (b) TG results for PVP (molecular weight of 58000) and the NS. In (a), major absorption peaks of PVP are found in the NS. In (b), the same range of weight losing temperature further indicates the existence of PVP in the NS. Taking into account of the moisture and residual, the content of PVP in the NS is calculated to be ca. 16.7%.

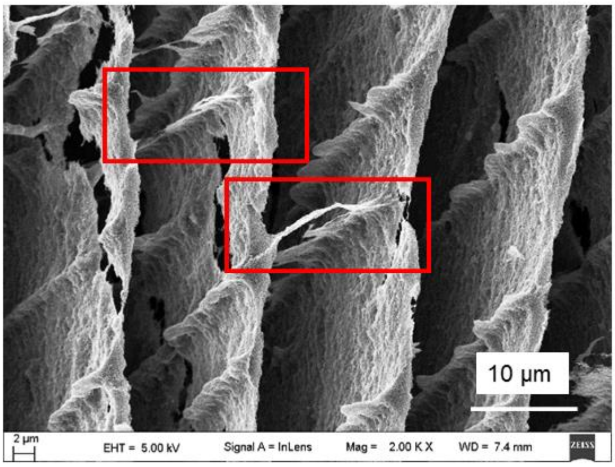

Figure S2. SEM images of the PCM for the cross section at high resolution. The red frames indicate the rib-like protrusions that structurally connect each lamella layer.

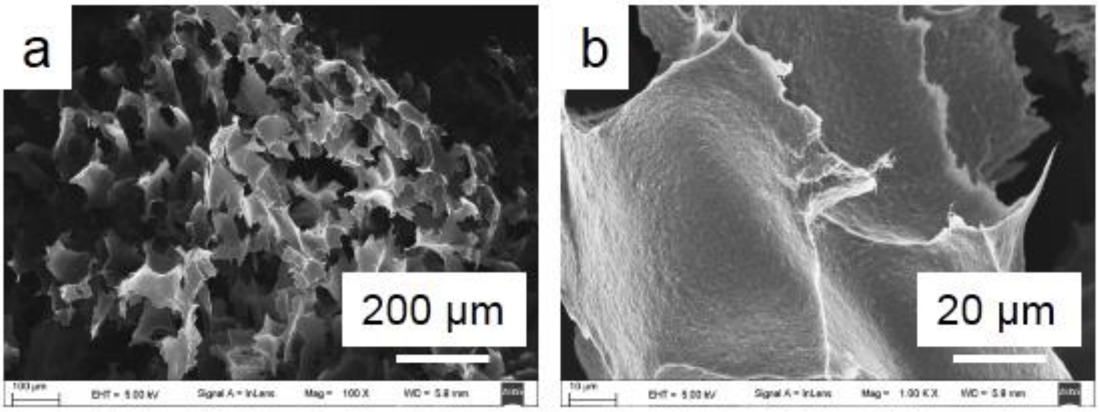

Figure S3. SEM images of the bulk sample prepared with the refrigerator freezing followed with freeze-drying at low magnification (a) and high magnification (b).

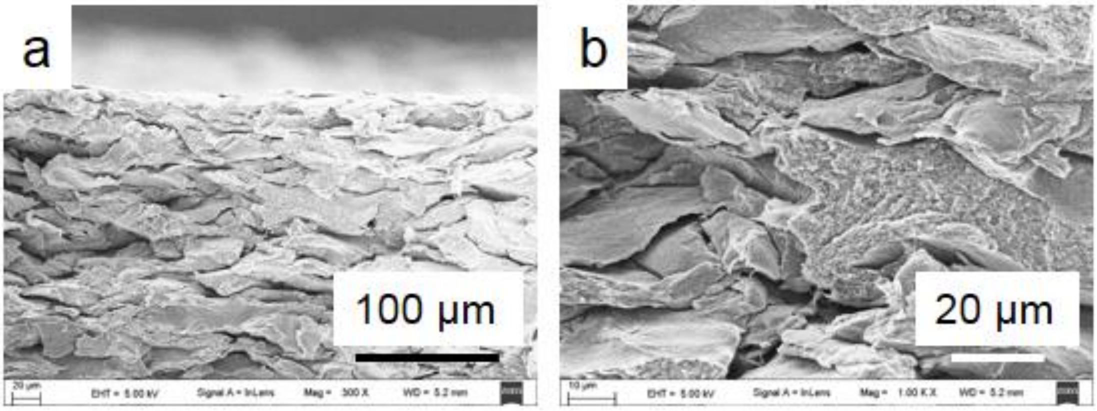

Figure S4. SEM images of the cross section of the RS at (a) low magnification and (b) high magnification.

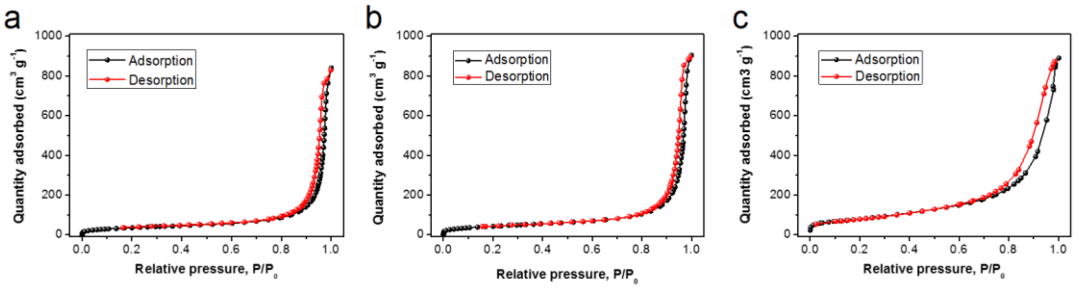

Figure S5. N2 adsorption/desorption isotherms for (a) NS, (b) RS and (c) CNT membrane. The measured SSAs of NS, RS and CNT membrane are 132, 157 and 236 m^2 g^{-1} and the corresponding pore volumes are 1.3, 1.4 and 1.4 cm^3 g^{-1}, respectively. Although the CNT

membrane has the largest SSA, it still shows the worst electrochemical performance because the CNT membrane cannot effectively hold the S.

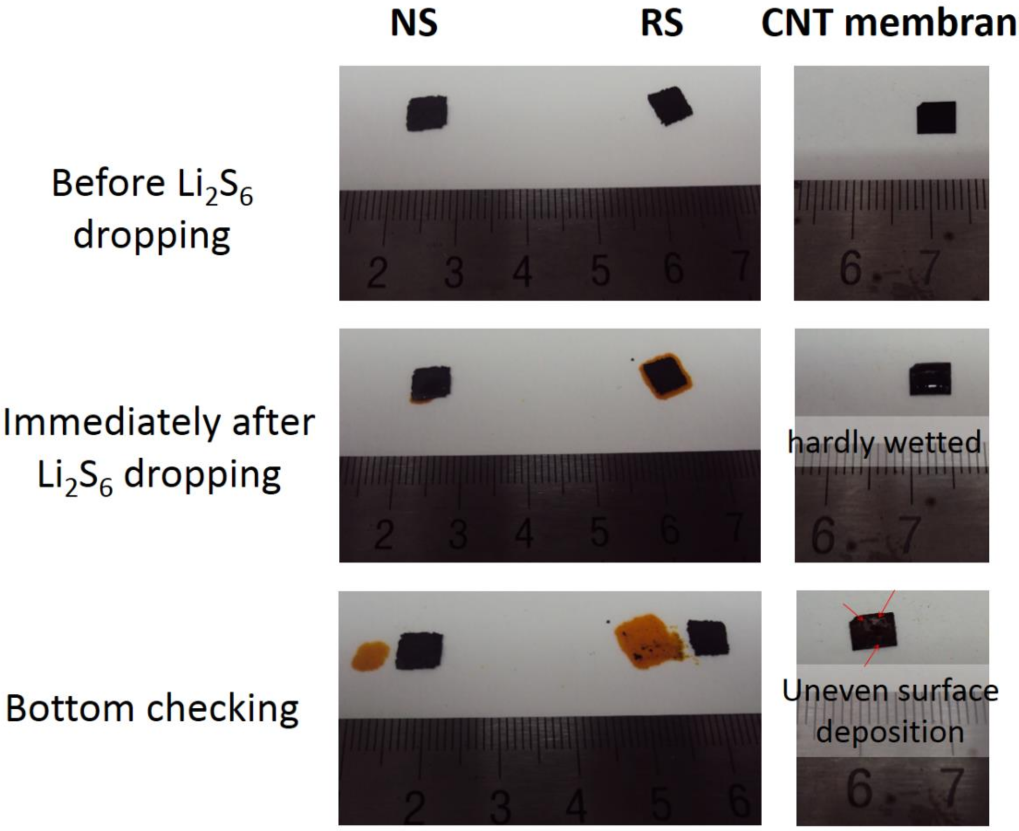

Figure S6. Photos for penetrating texts of NS, RS and a CNT membrane. For the NS and the RS, upon the catholyte dropping, the sheet adsorbs the liquid catholyte immidiately. On the other hand, the CNT membrane was harly wettable by the catholyte. Moreover, eneven solid deposition was observed after 5 min (bottom image). Thus, the presence of amphiphilic PVP is essential for a good wettability of the catholyte. The structural difference between the NS and the RS has rendered the different capability for accommodating the Li_2S_6 catholyte. The random porous structure of the RS turned out to be less capable for the catholyte accomodation than the micro-scale lamella structure of the NS. The better capacity of the NS is ascribed to the capillary force induced from the nano-interspace between layers[S5]. Note that all the sample pieces used here have almost the same weight of ca. 1.4 mg.

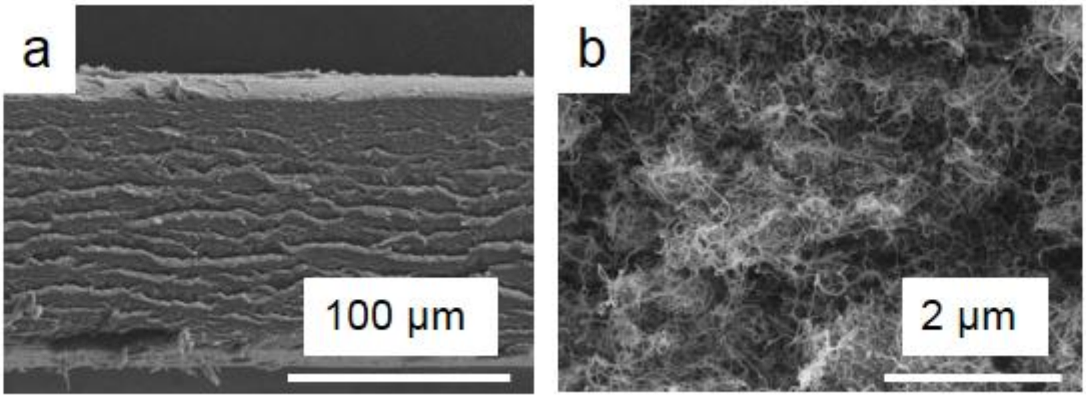

Figure S7. SEM images of the cross section of the CNT membrane at (a) low magnification and (b) high magnification.

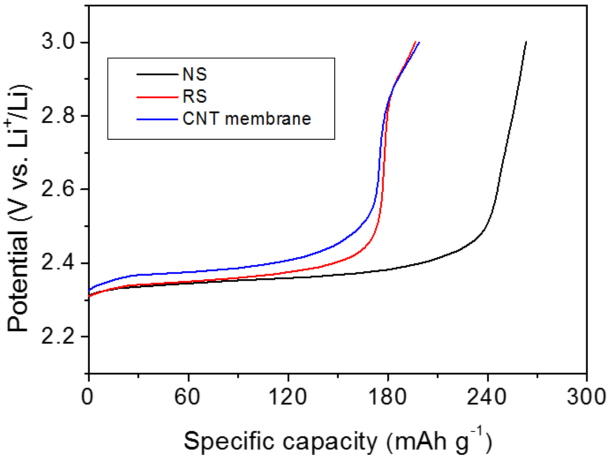

Figure S8. Galvanostatic charge profile for the initial pre-charging (delithiation) for the NS, RS and CNT membrane electrodes at 0.1 C up to 3.0 V. Consistent with other results, the RS and CNT membrane showed lower pre-charge capacity basically from the lose of Li_2S_6 upon cell assembling.

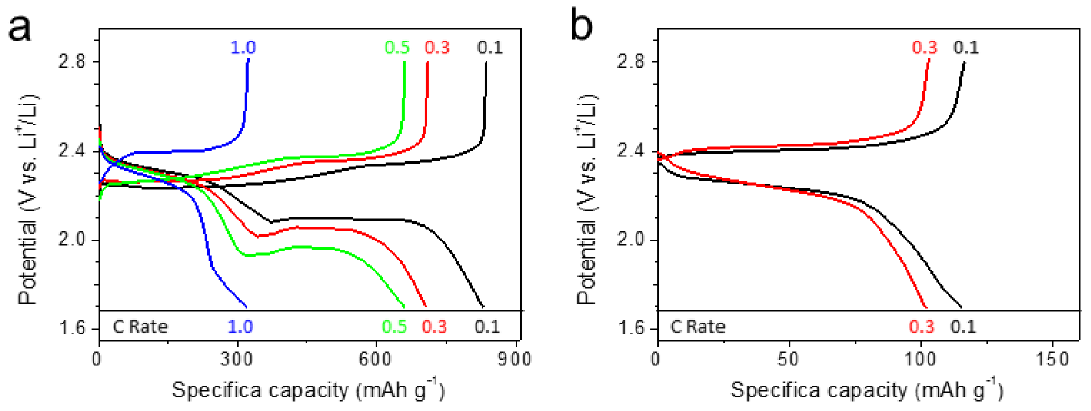

Figure S9. Galvanostatic charge/discharge profiles at different rates for the NS electrode with an areal sulfur loading of (a) 2 mg cm^{-2} and (b) 5 mg cm^{-2}. Sulfur was loaded by dropping ca.12.5 μL sulfur/carbon disulfide solution (100 mg mL^{-1}) into the NS, with subsequent drying up and a thermal treatment at 155 ºC.

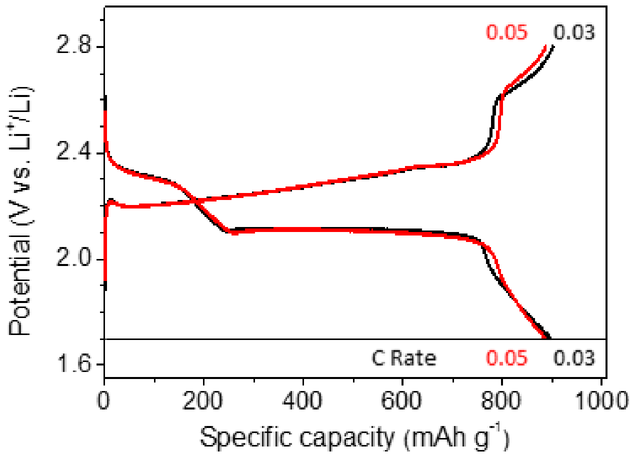

Figure S10. Galvanostatic charge/discharge profiles at low rates for the RS electrode with an areal sulfur loading 5 mg cm^{-2}. The discharge capacities at 0.03 C and 0.05 C are 897 and 886 mAh g^{-1}, respectively. These values are very close to the discharge capacity at 0.1 C (878 mAh g^{-1}).

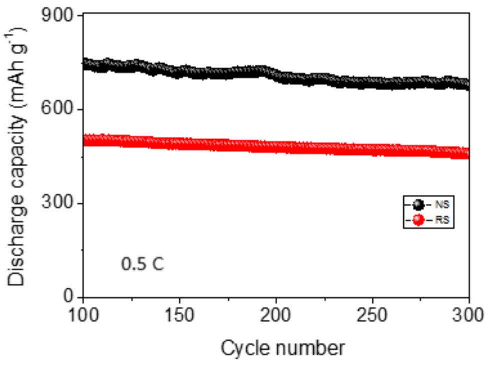

Figure S11. Enlarged graph of Fig. 2d for the discharge capacity in the lateral 200 cycles.

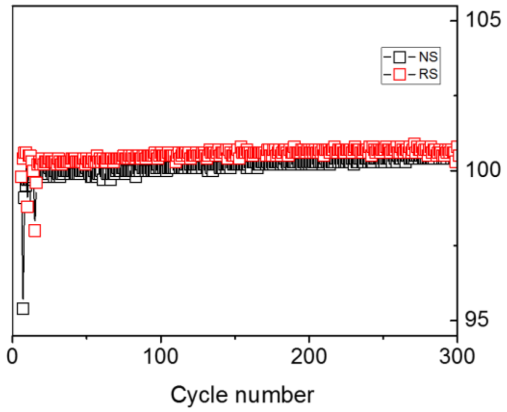

Figure S12. Coulombic efficiency for the NS and RS included cells with 5 mg cm^{-2} sulfur loading in a range of 95%-105%. Note that data for the reaction cycles are not included. Clearly shown in the curves, the Coulombic efficiency after stabilization for both the NS and RS electrodes are slightly higher than 100%.

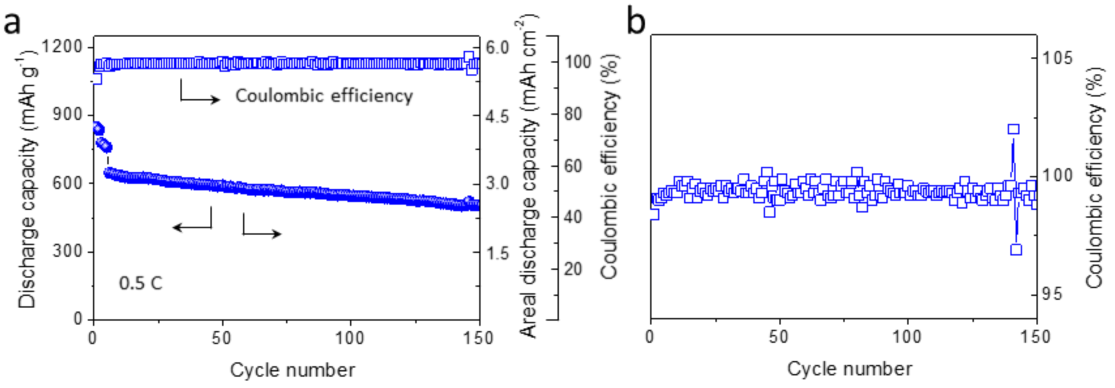

Figure S13. (a) Cycling performance tested at 0.5 C for the CNT membrane electrode (areal sulfur loading is ca. 5 mg cm^{-2}). (b) Coulombic efficiency for the RS included cell with a range of 95%-105%. Note that in (a), the initial 5 cycles refer to the activation cycles which include 2 cycles at 0.1 C and 3 cycles at 0.2 C, while in (b), the data for the activation cycles are not shown. The Coulombic efficiency is ca. 99.2% on average, with most cases below 100%. This is likely to be due to the lack of the capability of reutilizing the residual LiPSs without the anchoring cites provided by the amphiphilic PVP.

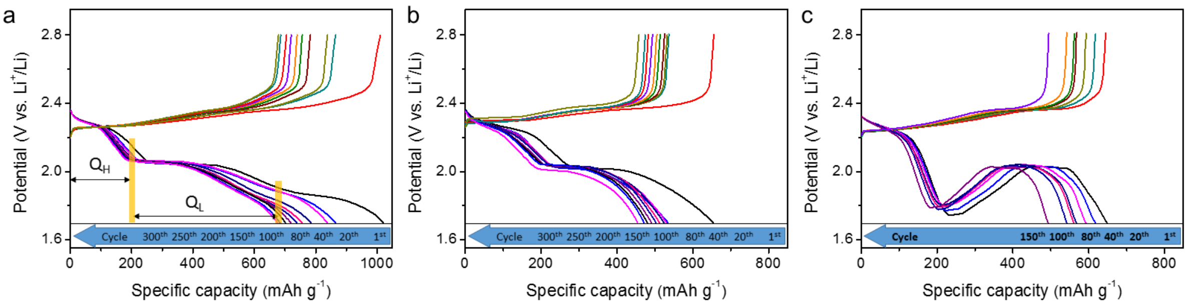

Figure S14. Galvanostatic charge/discharge profiles at a rate of 0.5 C over cycling for (a) the NS cathode, (b) the RS cathode and (c) the CNT membrane cathode. Q_H and Q_L are noted within the figure, corresponding to the different electrochemical processes that are involved. Q_H represents the electrochemical steps within the range of 2.4-2.1 V vs Li^+/Li, and Q_L represents the electrochemical steps thereafter and are within the range of 2.1-1.7 V vs Li^+/Li. For the NS and RS cathodes, cycle numbers of 1st, 20th, 40th, 60th, 80th, 100th, 150th, 200th, 250th and 300th are provided. For the CNT membrane cathode, cycle numbers of 1st, 20th, 40th, 60th, 80th, 100th and 150th are provided. Note that the curves for activation cycles are not provided here. Worth mentioning is the abnormal discharge cruves for the CNT membrane cathode, which might be ascribed to the relatively sever shuttling through cycling without the help of PVP.

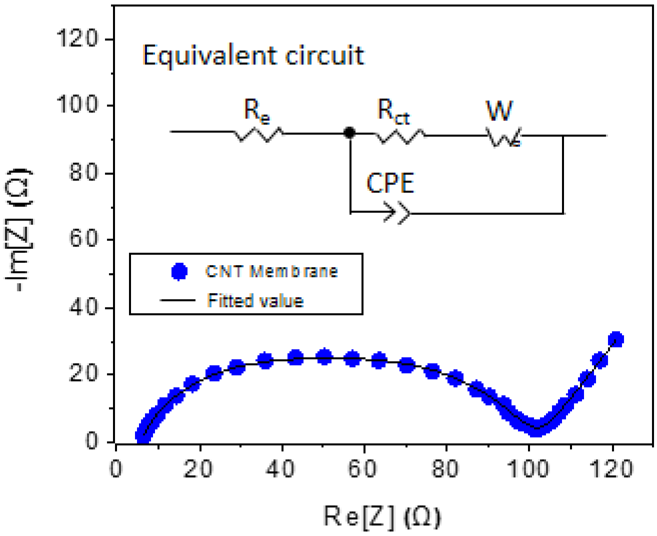

Figure S15. Nyquist plot with the fitted equivalent circuit for the CNT membrane electrode before cycling.

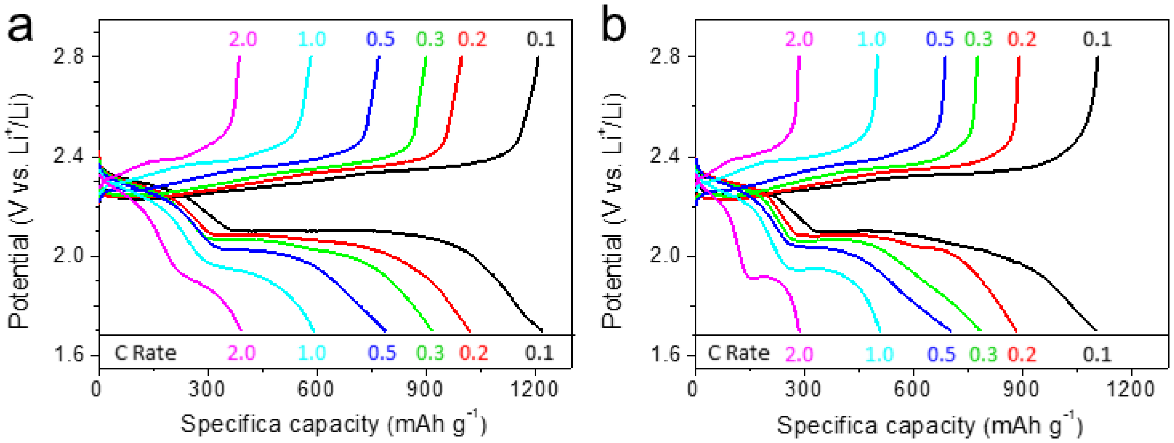

Figure S16. Galvanostatic charge/discharge profiles at rates from 0.1C to 2.0C for the NS with an areal sulfur loading of (a) 7.5 mg cm^{-2} and (b) 10 mg cm^{-2}.

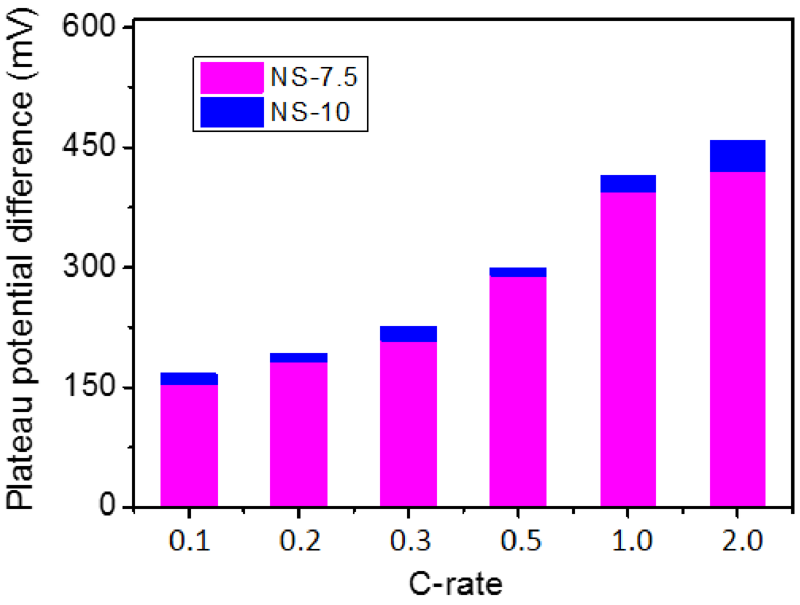

Figure S17. The voltage hysteresis for the NS-7.5 and NS-10 electrodes at different rates from 0.1 C to 2.0 C.

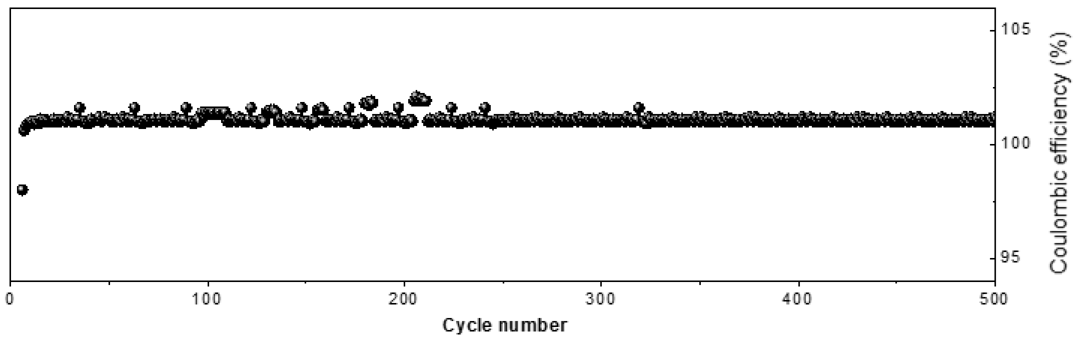

Figure S18. Coulombic efficiency for the NS-10 included cell with a range of 95%-105%. Note that data for the reaction cycles are not included. The cell shows a slightly higher Coulombic efficiency than the NS-5.0 included cell. This would also be due to the higher amount of LiPSs in the system.

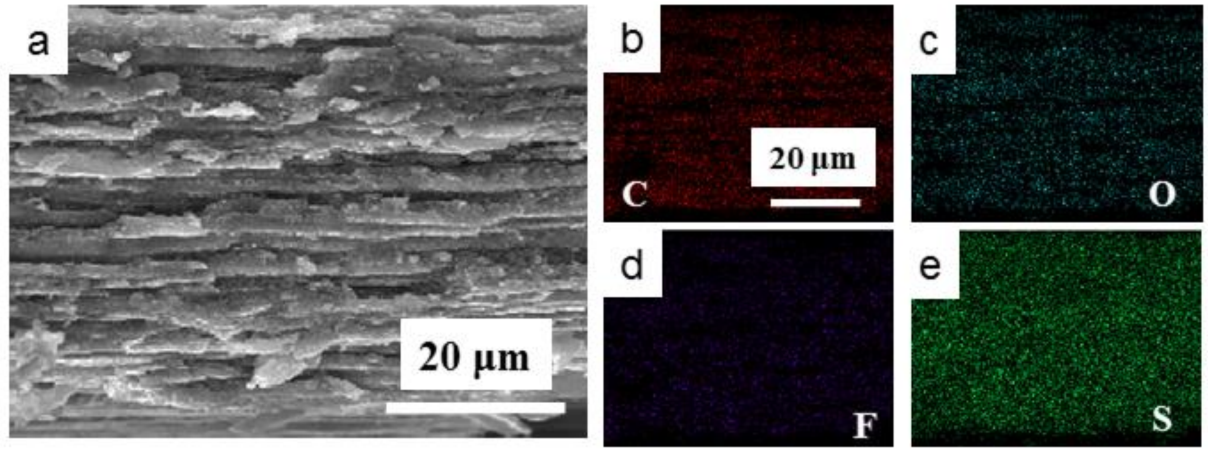

Figure S19. (a) SEM image of the NS electrode after 300 cycles. (b-e) Elemental mappings of C (b), O (c), F (d), and S (e) by EDS.

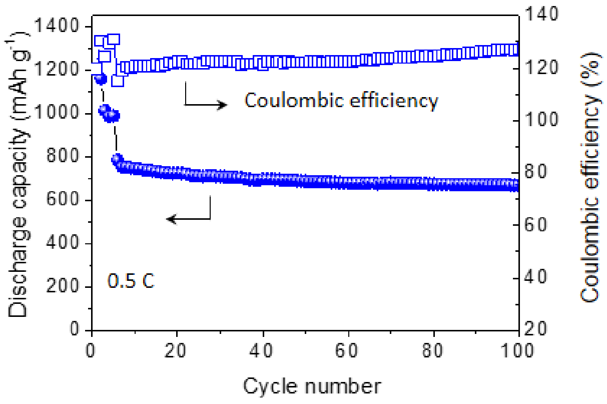

Figure S20. Cycling performance tested at 0.5 C for the NS electrode (areal sulfur loading is ca. 5 mg cm-2) in a $LiNO_3$-free system. Note that the initial 5 cycles refer to the activation cycles which include 2 cycles at 0.1 C and 3 cycles at 0.2 C. The discharge capacity is very close to that in the $LiNO_3$-containing system. The abnormally high Coulombic efficiency might be due to the decomposition of the electrolyte components such as 1,3 –dioxolane, which contributes to the charge capacity[S6].

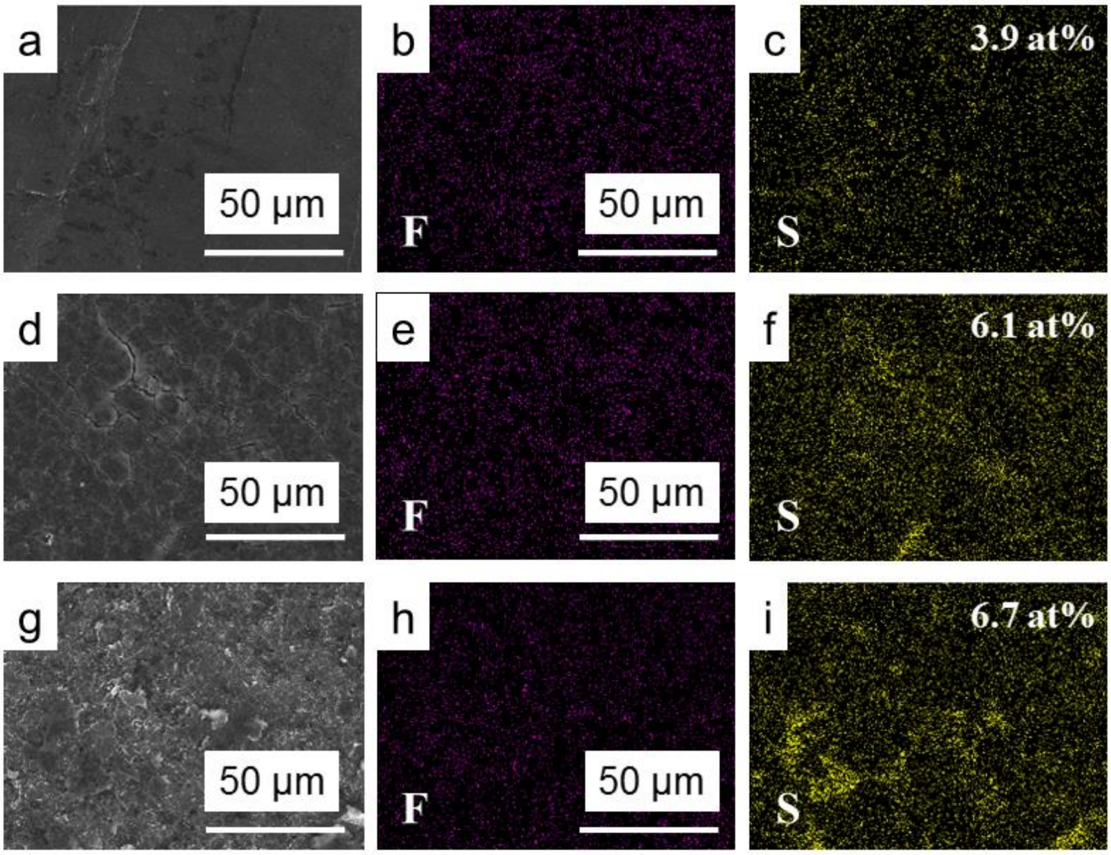

Figure S21. SEM images and elemental distribution of the lithium metal surface after 50 charge/discharge cycles with the (a-c) NS electrode, (d-f) RS electrode and (g-i) CNT membrane electrode. It was found that the S content is ca. 3.9 at% on the lithium metal surface with the NS electrode after 50 cycles. This S content is much less than that with the RS electrode and the CNT membrane electrode (ca. 6.1 at% and ca. 6.7 at%, respectively), indicating that the NS electrode can more effectively restrain the polysulfide dissolution and the shuttling than the RS electrode and the CNT membrane electrode.

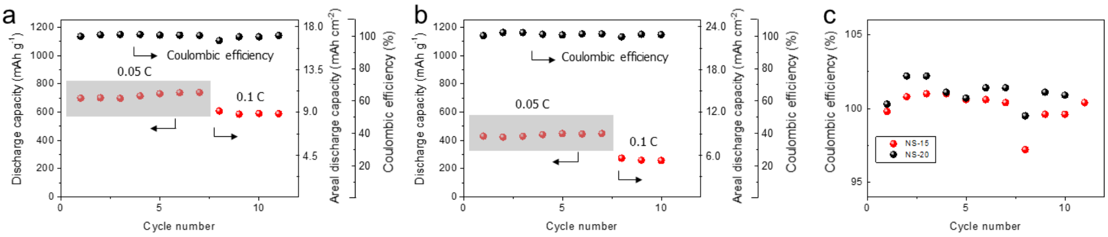

Figure. S22. Cycling test for (a) the NS-15 and (b) NS-20 (corresponding to an areal sulfur loading of 15 mg cm^{-2} and 20 mg cm^{-2}) electrode. (c) Coulombic efficiency for the NS-15 and

NS-20 included cells with a range of 95%-105%. The corresponding cells were assembled using a commercially available PP separator coated with a thin layer of carbon, and the weight for the NS used was ca. 2.0 mg. Note that the initial 2 cycles at 0.05 C refer to the activation cycles.

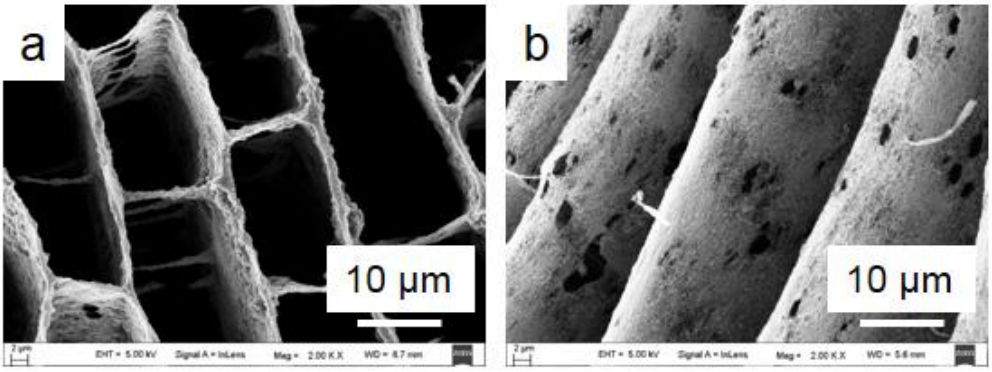

Figure S23. SEM images of the NS for the cross section along the length direction prepared from an aqueous CNT dispersion with a solid content of (a) ca. 3.6 wt% and (b) 1.2 wt%. The immersing velocity used was 50 cm h^{-1}. The average thickness of the channel wall in (a) is ca. 730 nm, while that in (b) was too thin that the channel walls easily bend when the electron beam irradiates on them.

Table S1. Electrochemical performance of high-sulfur loading cathodes.

Cathodes (Form of sulfur)	Areal sulfur loading (mg cm^{-2})	Sulfur content (wt%)	Capacity at 0.2 C[a] (mAh g^{-1})	Capacity at 1.0 C (mAh g^{-1})	Capacity retention	Refs
N/S-doped CNT-graphene hybrid (Li_2S_6)	4.6	63~72.5	1157	675	63% at 0.5C for 500 cycles	S6
Graphene encapsulated hollow mesoporous carbon nanosheet (S)	5.0	73	1050	733	73.8% at 0.2C for 50 cycles	S7
Co(OH)2@LDH nanocages (S)	3.0	52.5	800	500	60% at 0.5C for 100 cycles	S8
N-doped CNT-Graphene 3D nanostructure (S)	4.2-4.4	64	N.A.[b]	806	96% at 2C for 200 cycles[c]	S9
NbS2@S@iodine-doped graphene (S)	3.25	67.5	811	N.A.	80% at 1C for 600 cycles[d]	S10
MoO_2@CNT+activated carbon nanofibers (Li_2S_6)	4.8	55	826	627	87% at 0.5C for 100 cycles	S11
Carbon nanofiber (S)	5.7	52.9	1187	782	62% at 0.2C for 200 cycles	S12
S@Graphene (S)	4.32	90	772	500	65.4% at 0.1C for 50 cycles	S13
NS	5	54	1156	958	66.3% at 0.5 C for 300 cycles	★This work

[a] 1 C=1675 mAh g^{-1}. [b] Not available. [c] The value is based on the 2nd cycle. [d] The results involve a capacity increase in the initial 100 cycles.

Table S2. Impedance values of the equivalent circuit fitted for the impedance spectra for the NS, RS and CNT membrane electrodes.

Sample	R_e (Ω)	R_{ct} (Ω)	W_o (Ω)
NS	6.9	62.5	22.6
RS	4.2	36.1	30.1
CNT membrane	5.3	91.7	40.1

Supplementary references

S1. Nishihara, H., Mukai, S.R., Yamashita, D. & Tamon, H. Ordered macroporous silica by ice templating. *Chem. Mater.* **17**, 683-689 (2005).

S2. Pan, Z.-Z. *et al.* Cellulose nanofiber as a distinct structure-directing agent for xylem-like microhoneycomb monoliths by unidirectional freeze-drying. *ACS nano* **10**, 10689-10697 (2016).

S3 . Mukai, S.R., Nishihara, H. & Tamon, H. Formation of monolithic silica gel microhoneycombs (SMHs) using pseudosteady state growth of microstructural ice crystals. *Chem. Commun.* **10**, 874-875 (2004).

S4. Niu, S. *et al.* A carbon sandwich electrode with graphene filling coated by N-doped porous carbon layers for lithium–sulfur batteries. *J. Mater. Chem. A* **3**, 20218-20224 (2015).

S5. Lukatskaya, M.R. *et al*. Ultra-high-rate pseudocapacitive energy storage in two-dimensional transition metal carbides. *Nat. Energy* **2**, 17105 (2017).

S6. Tikerkar, M.D. *et al.* Design principles for electrolytes and interfaces for stable lithium-metal batteries. *Nat. Energy* **1**, 16114 (2016).

S7. Zhou, G., Paek, E., Hwang, G.S. & Manthiram A. Long-life Li/polysulphide batteries with high sulphur loading enabled by lightweight three-dimensional nitrogen/sulphur-codoped graphene sponge. *Nat. Commun.* **6**, 7760 (2015).

S8. Pei, F. *et al.* Self-supporting sulfur cathodes enabled by twodimensional carbon yolk-shell nanosheets for highenergy-density lithium-sulfur batteries. *Nat. Commun.* **8**, 482 (2017).

S9. Zhang, J., Hu, H., Li, Z., Lou, X.W.D. Double-Shelled Nanocages with Cobalt Hydroxide Inner Shell and Layered Double Hydroxides Outer Shell as High-Efficiency Polysulfide Mediator for Lithium–Sulfur Batteries. *Angew. Chem. Int. Ed.* **55**, 3982-3986 (2016).

S10. Ding, Y.-L. et al. Facile Solid-State Growth of 3D Well-Interconnected Nitrogen-Rich Carbon Nanotube–Graphene Hybrid Architectures for Lithium–Sulfur Batteries. *Adv. Funct. Mater.* **26**, 1112-1119 (2016).

S11. Xiao, Z., et al. Sandwich-Type NbS_2@S@I-Doped Graphene for High-Sulfur-Loaded, Ultrahigh-Rate, and Long-Life Lithium−Sulfur Batteries. *ACS nano* **11**, 8488-8498 (2017).

S12. Xu, H., Qie, L., Manthiram., A. An integrally-designed, flexible polysulfide host for high-performance lithium-sulfur batteries with stabilized lithium-metal anode. *Nano Energy* **26**, 224-232 (2016).

S13. Qie, L, Manthiram., A. A Facile Layer-by-Layer Approach for High-Areal-Capacity Sulfur Cathodes. *Adv. Mater.* **27**, 1694-1700 (2015).
